# An untapped window of opportunity for glioma: targeting therapy-induced senescence prior to recurrence

**DOI:** 10.1038/s41698-023-00476-8

**Published:** 2023-11-29

**Authors:** Cecile Riviere-Cazaux, Lucas P. Carlstrom, Bryan J. Neth, Ian E. Olson, Karishma Rajani, Masum Rahman, Samar Ikram, Moustafa A. Mansour, Bipasha Mukherjee, Arthur E. Warrington, Susan C. Short, Thomas von Zglinicki, Desmond A. Brown, Sandeep Burma, Tamar Tchkonia, Marissa J. Schafer, Darren J. Baker, Sani H. Kizilbash, James L. Kirkland, Terry C. Burns

**Affiliations:** 1 Department of Neurological Surgery, Rochester, MN USA; 2 Department of Neurology, Rochester, MN USA; 3 Department of Neurological Surgery, Northwestern University, Chicago, IL USA; 4 Department of Neurosurgery, University of Texas Health Science Center at San Antonio, San Antonio, TX USA; 5 Leeds Institute of Medical Research at St. James’s, St. James’s University Hospital, University of Leeds, Leeds, UK; 6 Biosciences Institute, Faculty of Medical Sciences, Campus for Ageing and Vitality, Newcastle University, Newcastle upon Tyne, UK; 7 National Institute of Neurological Disorders and Stroke, National Institutes of Health, Bethesda, MD USA; 8 Department of Physiology and Biomedical Engineering, Rochester, MN USA; 9 Department of Pediatric and Adolescent Medicine, Rochester, MN USA; 10 Department of Biochemistry and Molecular Biology, Rochester, MN USA; 11 Department of Medical Oncology, Rochester, MN USA; 12 Department of Medicine, Rochester, MN USA

**Keywords:** CNS cancer, CNS cancer

## Abstract

High-grade gliomas are primary brain tumors that are incredibly refractory long-term to surgery and chemoradiation, with no proven durable salvage therapies for patients that have failed conventional treatments. Post-treatment, the latent glioma and its microenvironment are characterized by a senescent-like state of mitotic arrest and a senescence-associated secretory phenotype (SASP) induced by prior chemoradiation. Although senescence was once thought to be irreversible, recent evidence has demonstrated that cells may escape this state and re-enter the cell cycle, contributing to tumor recurrence. Moreover, senescent tumor cells could spur the growth of their non-senescent counterparts, thereby accelerating recurrence. In this review, we highlight emerging evidence supporting the use of senolytic agents to ablate latent, senescent-like cells that could contribute to tumor recurrence. We also discuss how senescent cell clearance can decrease the SASP within the tumor microenvironment thereby reducing tumor aggressiveness at recurrence. Finally, senolytics could improve the long-term sequelae of prior therapy on cognition and bone marrow function. We critically review the senolytic drugs currently under preclinical and clinical investigation and the potential challenges that may be associated with deploying senolytics against latent glioma. In conclusion, senescence in glioma and the microenvironment are critical and potential targets for delaying or preventing tumor recurrence and improving patient functional outcomes through senotherapeutics.

## Introduction

**Fig. 1:**
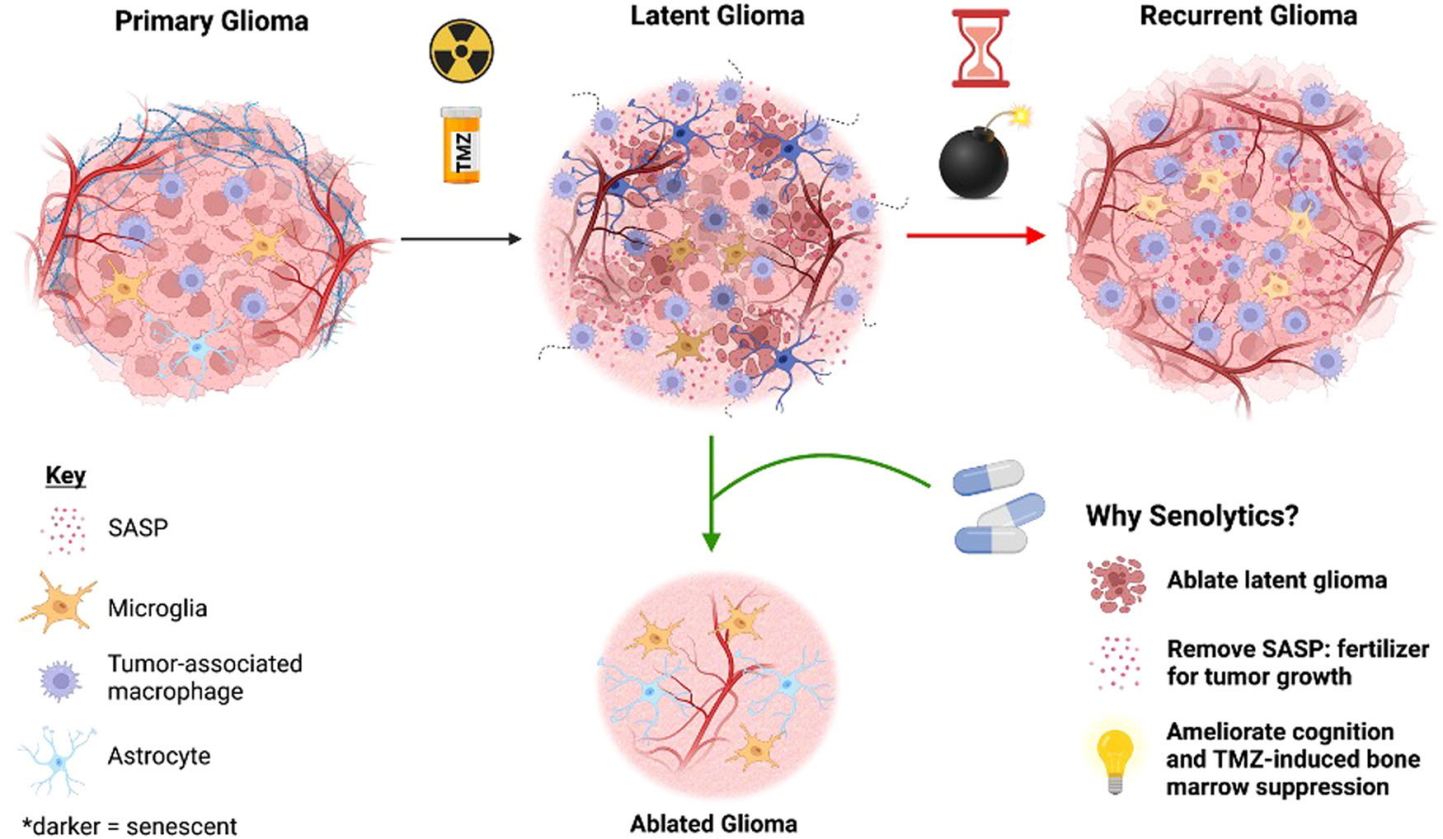
Senescence in latent glioma. Primary gliomas contain non-senescent microglia, tumor-associated macrophages, and minimal abundance of the senescence associated secretory phenotype (SASP). After chemoradiation, the latent glioma microenvironment is characterized by senescence of each of these components of the tumor microenvironment (TME) as well as the injured glioma cells, along with increased SASP components. Currently, no treatments exist for latent glioma, leading to inevitable tumor recurrence characterized by abundant tumor-associated macrophages, tumor invasion, and aggressivity. Senolytics in latent glioma may be of use to ablate these latent tumor cells and the microenvironment that contribute to aggressive glioma recurrence. Created using BioRender.

Gliomas are primary brain tumors, the most aggressive of which is glioblastoma, which has a dismal median survival of only 15 months^1^. Despite maximal safe surgical resection and aggressive radiation and temozolomide (TMZ), all gliomas inevitably recur, typically within the prior treatment field, and often more aggressively than the original tumor^2^. Since surgery is typically only performed in the context of newly diagnosed or recurrent disease, relatively little is known about the molecular characteristics and potential drug sensitivity of the latent human glioma cells that contribute to recurrence^3^. After completion of chemoradiation, no standard or experimental drugs exist to specifically target the latent glioma. Nevertheless, accumulating evidence suggests potential therapeutic vulnerabilities within the senescent-like resistant cell population surviving after chemoradiation in gliomas^4^ and other cancers^5^. Rather than waiting for radiographic and clinical tumor recurrence, targeting latent glioma cells may provide a unique opportunity for targeting senescent tumor cells to forestall, if not prevent, otherwise inevitable recurrence (Fig. 1).

After induction of DNA damage by chemoradiation, cells upregulate cell cycle regulatory factors, including p16, p53, and p21 to promote DNA repair, apoptosis, or senescence^6^. Senescence is a state of cell-cycle arrest characterized by an altered transcriptional, metabolic, and secretory phenotype (termed the senescence associated secretory phenotype, or SASP)^7^. Although senescence is intrinsically anti-tumorigenic by preventing uncontrolled cell proliferation, recent data suggest that it may also play an important role in glioma recurrence^8,9^ through multiple mechanisms. First, the acquisition of senescence allows for cells to escape therapy-induced apoptosis^10–12^. Although previously considered irreversible, recent evidence suggests that this latent population of malignant cells could potentially “escape” senescence after treatment completion, causing cell cycle re-entry and tumor recurrence^8^. As such, while paradoxical to the definition of senescence as a state of permanent cell-cycle arrest, this review will utilize the term “senescent” tumor cells to describe glioma cells that have entered a senescent-like state of treatment-induced mitotic arrest prior to escaping senescence and re-entering the cell cycle. Second, radiation-induced senescence in the glioma microenvironment has been shown to accelerate tumor growth, likely mediated by the proinflammatory and preremodeling SASP, with outcomes ameliorated by clearance of senescent stromal cells^13^. Finally, senescence is also increasingly implicated as a targetable pathogenic mechanism in radiation-induced injury, chemotherapy-induced bone marrow suppression, and neurodegenerative diseases^14–17^. Numerous drugs are being developed or repurposed as senolytics to specifically target senescent cells^18^. As such, directed treatment for latent gliomas after conventional chemoradiation may provide an opportunity to ablate senescent cells prior to tumor recurrence while also ameliorating the side effects of therapy.

In this review, we discuss the potential implications of senescence to glioma therapeutics. Of note, this will be discussed in the broad diagnosis of “glioma” rather than specifying the glioma subtype according to the CNS WHO 2021 criteria^19^. Most preclinical and clinical studies have been performed in models of isocitrate dehydrogenase (IDH)-wild type glioblastoma as defined by CNS WHO 2021 criteria, with a select few studies in grade 4 IDH-mutant astrocytoma models. There is currently insufficient evidence to suggest a difference in the senescent tumor or microenvironment burden across glioma subtypes. However, treatment-induced senescence is likely applicable to all glioma subtypes that have undergone chemotherapy or radiation. As such, this review intentionally utilizes the overarching diagnosis of “glioma,” except when specifying the models utilized in the studies.

Evolving data suggest that targeting senescence may (1) ablate latent tumor cells that could contribute to tumor recurrence, (2) remove SASP factors from the tumor microenvironment that could otherwise promote aggressive glioma recurrence, and (3) alleviate the long-term deleterious impacts of prior therapies on cognition and chemotherapy-induced bone marrow suppression. We evaluate currently available senolytics and data available for targeting latent glioma with senolytic drugs (Table 1). Finally, we discuss challenges and opportunities for translation of senolytics into the clinic that may impact clinical trial design and selection of relevant outcome measures. We highlight the potential of senescent cell ablation in latent gliomas and their microenvironment as a rational, but underexplored, window of opportunity to accelerate therapeutic progress against this notorious disease.

**Table 1:** Selection of candidate senolytic/senomorphic therapies in glioma.

Drug family	Senotherapeutic (FDA status)	Mechanism	In vitro and in vivo evidence
Anti-apoptotic Bcl-2 family protein inhibitors	ABT-263/ navitoclax (in clinical trials)	Bcl-2, Bcl-xL, and Bcl-W inhibitor	+ Apoptosis induced at lower IC50 in senescent, than non-senescent, glioma cells across multiple cell lines^102–104^. + Increased animal survival in pre-irradiated in vivo CT-2A;^13^ also in radiation-naïve U87MG IDH1-R132H and PDX GBM164 models^103^. -Drug toxicities, including thrombocytopenia^106^.
	Venetoclax (FDA approved)	Bcl-2 inhibitor	+ Venetoclax after initial round of TMZ increases cell death in in vitro LN229^108^. - No differential impact of drug on senescent human glioma cells as compared to non-senescent cells^104^.
Flavonoids	*Quercetin*, in combination with dasatinib (in clinical trials^53^)	SCAP targeting, including tyrosine kinases (dasatinib), PI3K/AKT/mTOR, p53/MDM2/p21 and HIF1${\rm{\alpha }}$- related pathways (quercetin)	+ In other disease models, decrease in circulating SASP factors, and clearance of senescent cells in adipose tissue^15,53,109^. - No substantial impact on senescent in vitro GBM39 human glioma cells^104^.
	Fisetin (in clinical trials)	Inhibition of multiple pathways, including PI3K/AKT/mTOR and AMPK	+ Decrease in senescent cells after TMZ-induced senescence in LN229 and A172 cells^102^. - No senolytic impact on senescent in vitro GBM6, 39, or 76 cells^104^.
Inhibitors of apoptosis (IAP) family inhibitor	BV6 (not available clinically)	Inhibition of c-IAP1 and c-IAP2	+ Selectively ablate in vitro LN229 and A172 after TMZ^93^. + Combination with venetoclax and TMZ increase cell death as compared to BV6, venetoclax, +/- TMZ^99^.
Antimalarial	Artesunate	Poorly defined	+ Senolytic activation in TMZ-treated LN229, A172, and U87MG in vitro cells^86^, as well as GBM stem-like cells^99^.
Hsp90 inhibitors	Onalespib (in clinical trials^99^)	Inhibitor of Hsp90	+ In combination with TMZ, increased survival in intracranial U251 and GS811 mouse intracranial xenografts as compared to TMZ alone^117^. +/- Cytotoxic impact in GBM6, 39, and 76, albeit not specific to senescent cells^104^.
Senomorphics, i.e SASP inhibition	Tocilizumab (FDA approved)	IL-6 therapeutic monoclonal antibody	+ Decreased growth of TMZ-treated subcutaneous flank U251 glioma^121^. + Targeting IL6R-alpha or IL-6 with shRNA decrease glioma stem cell growth^71^. + IL-6/IL-6R knockdown increase survival in intracranial T3359 GSC models^71^.

## The deleterious impacts of senescence in glioma

### Defining senescence and senolytics

Cellular senescence is an irreversible state of cell-cycle arrest characterized by a pro-inflammatory senescence-associated secretory phenotype (SASP)^20^. This state has been most extensively characterized in aging and non-glioma diseases. SASP factors include cytokines and chemokines such as IL-6^21^, IL-8^22^, and MIP-3α^23^, in addition to growth factors^24^ and matrix metalloproteases^25,26^. While senescence has mainly been studied in the context of aging, it can also be induced by injurious cellular stressors to protect the cell from further harm or malignant transformation. Senescence may be induced by DNA damage, viral infection, oxidative stress, telomeric dysfunction, and other previously reviewed mechanisms^27,28^. Upregulation of the cyclin-dependent kinase inhibitors p16 and p21 contributes to cellular senescence by preventing cell cycle progression from the G_1_ to S phase^29^. p16 (also known as p16^INK4A^) is one of multiple proteins encoded by Cdkn2A along with p14 (p14^ARF^, human) or p19 (p19^ARF^, mouse) which stabilize p53 by preventing its destruction through inhibiting Mdm2 activity^30^. p21 (p21^CIP1^; p21^WAF1^) is encoded by Cdkn1a and mediates senescence in response to p53 and p53-independent stimuli, including NF-kB and TGF-β signaling^31,32^. Indeed, we have recently shown that p21 overexpression promoted a senescent phenotype in multiple human glioma cell lines in a more stable and efficient manner than radiation^33^.

It is crucial to acknowledge the inherent diversity and variability in cellular senescence. Indeed, senescent cells are identified by multiple markers rather than a singular unifying one^34^. Particularly, the composition of SASP, which plays a pivotal role in determining the characteristics of senescent cells, is highly context-dependent, influenced by factors such as the cell-of-origin^22^, environment immune status (previously reviewed^35,36^), and induction via normal aging versus disease versus treatment (previously reviewed^37,38^). A prior study in human fibroblasts utilized single-cell isolation and nanofluidic PCR techniques to conclude that senescent gene expression signatures were highly variable from one senescent cell to another^39^. Specifically, SASP-encoding genes varied significantly across senescent cells, with only a subset of genes being consistently upregulated across the population. Moreover, senescence and SASP can exhibit dynamic changes over time, particularly regarding the time since induction of senescence after therapy. The four states induced after induction of senescence, including initiation, early, full, and late senescence, each have unique characteristics based on metabolic and epigenetic remodeling, as previously reviewed^40^. To our knowledge, no study has extensively evaluated the changes in senescence and SASP over time in glioma, particularly as a function of time since treatment.

Cellular senescence is a potent inhibitor of tumorigenesis within damaged cells, preventing the propagation of compromised genetic material and uncontrolled progression through the cell cycle^41^. However, senescence and its induced SASP factors can also contribute to a deleterious inflammatory and degradative state in the surrounding microenvironment^42^. Senescence has been implicated in a growing number of aging-associated diseases including dementia^43^, arthritis^44^, osteoporosis^45^, atherosclerosis^46^, and frailty^47^. In these conditions, senescent cells accumulate with prolonged age in tissues and organs, resulting in local and systemic dysfunction. Elimination of senescent cells through either genetic or pharmacological (senolytic) means improves tissue function and animal longevity in preclinical models of aging and degenerative disease^15,48^. Transplanting senescent cells into younger mice accelerated frailty and aging-associated deaths^49^. Senescent cells typically rely on anti-apoptotic pathways termed “senescent cell anti-apoptotic pathways” (SCAPs) for survival^50^. A growing repertoire of senolytic drugs, including dasatinib and quercetin^51^, have been found to target SCAPs, leading to selective apoptosis of senescent cells^52^. Clinical trials for several senescence-associated diseases are currently underway, with early results demonstrating efficacy in decreasing the human senescent cell burden^53^.

Recent studies suggest that senescent cells may be present in primary gliomas. One study identified senescent cells in primary glioblastoma patient samples, as well as in a GLAST^CreERT2/+^;Pten^fl/fl^ mouse glioblastoma model^54^. Partial removal of senescent cells using the *p16-3MR* transgenic mouse model via ganciclovir or treatment with navitoclax (ABT263), an inhibitor of Bcl-2 and Bcl-xL, prolonged survival by 28–35% (10–12 days) from time of tumor implantation. It remains unknown how this relative senescent burden at baseline is impacted after standard glioma therapy based on longitudinal samples.

### Therapy-induced senescence in glioma

Temozolomide (TMZ) is an alkylating chemotherapy that induces senescence in malignant glioma cells by triggering an O^6^-methylguanine (O^6^MeG) DNA lesion which results in an accumulation of senescent cells at the G2-M phase of the cell cycle^55^. This TMZ-induced senescent state occurs with similar kinetics to apoptosis and is characterized by double-strand DNA breaks (DSBs), as well as increased ROS-induced DNA damage^56^. Sensitivity to TMZ is impacted by MGMT (O^6^-methylguanine-DNA methyltransferase) methylation status, wherein MGMT methylation epigenetically silences expression of this DNA repair enzyme, rendering these tumors more susceptible to alkylating agents and improving prognosis after chemoradiation as compared to MGMT-unmethylated patients^57,58^. Using a Tet-on system to activate MGMT at various timepoints from TMZ exposure, one group demonstrated that O^6^MeG was required for the induction, but not the maintenance, of this post-TMZ senescent state^56^. Aasland et al. also found that in addition to dependence on p53, complete knockdown of p21 abrogated the induction of senescence, indicating that TMZ-induced senescence is dependent on p21^59^. Senescence-associated cell-cycle arrest has classically been considered irreversible through the accumulation of p14/p16 and p21. TMZ-associated senescence induces the SASP in an NF-κB dependent manner and represses the transcription of mismatch repair and homologous recombination proteins, such as *EXO1, MSH2, MSH6*, and *RAD51*^59^.

Radiation leads to DNA damage by inducing oxidative stress and double-strand breaks as well as abasic sites. This severe DNA damage induces a senescent state in glioma cells that do not undergo apoptosis and are undetectable radiographically. Radiation-induced senescence has been identified in numerous glioblastoma cell lines, including LN229^60^, the p53 wild-type U87 line^61,62^, various PTEN-deficient cells such as U251 and U373^61^, and numerous human glioblastoma-derived cell lines within a week of irradiation^33^. The presence of a senescent phenotype was indicated in these cases by senescence-associated β-galactosidase (SA-β-Gal) staining, characteristic enlarged and flattened cellular morphology characteristic of senescent cells, SASP production and/or mitotic arrest. Another study demonstrated widespread non-tumoral murine astrocyte senescence after brain irradiation as evidenced by increased CDKN1A, SA-β-Gal staining, and elevated SASP factors, including hepatocyte growth factor (HGF), underscoring the relevance of radiation as a SASP-inducing agent^13^. Interestingly, a recent study suggested that while radiation-induced senescence in both male and female glioblastoma xenograft mice, there was a greater post-irradiation senescent cell burden in the stromal cells of female mice based on SA-β-Gal staining and p21 expression^63^.

Of note, it remains unknown whether there is a difference in therapy-induced senescence across glioma subtypes, such as astrocytoma versus oligodendroglioma, low-grade versus high-grade, and across molecular characteristics (IDH-wild type versus mutant, MGMT methylated versus unmethylated). This is in part due to preclinical models that focus on glioblastoma, with a few models available for grade 4 IDH-mutant astrocytoma, in addition to a lack of characterization of senescence across diverse human glioma tissue samples, especially after treatment. We speculate that glioma subtypes that have longer periods of disease latency after treatment, such as low-grade gliomas or MGMT-methylated glioblastoma, may have a higher burden of senescent cells than gliomas that recur more rapidly, such as MGMT-unmethylated glioblastoma. Moreover, based on intratumoral glioma heterogeneity, it is also possible that there may be variability in senescence across glioma subtypes. An added difficulty is the lack of a clear consensus definition on how to identify senescent cells; rather than relying solely on one assay, such as SA-β-gal staining or p16 expression, multiple markers must be used. As such, further studies are needed to extensively characterize the relative burden of tumor and microenvironment senescence across glioma subtypes from human tissues obtained after treatment.

### The senescent tumor microenvironment (TME) impacts glioma aggressiveness

Although therapy-induced senescence is effective at transiently inducing cell-cycle arrest, recurrence inevitably occurs. Unfortunately, despite the anti-tumor properties of senescence, recurrent tumors are often more aggressive and resistant to further therapy than the original treatment-naïve disease^64^. Understanding why and how this occurs may reveal novel therapeutic opportunities, particularly regarding the relative contribution of cells escaping senescence versus SASP-induced aggressiveness of recurrent tumor cells that were never senescent. As described in “The senescent escape” below, a small subset of senescent tumor cells may escape senescence and contribute directly to glioma recurrence. However, most senescent cells are likely to persistently remain in this latent state within the tumor microenvironment. As such, the impacts of the senescent tumor microenvironment (TME) described in this section could be induced either by cells which are transiently or persistently senescent. However, we speculate that the latter are more likely to contribute overall to glioma aggressiveness over time as they persist in the latent microenvironment and release SASP.

The senescent tumor microenvironment (TME), induced by chemoradiation and characterized by increased SASP, may contribute to glioma recurrence. The SASP itself can be proangiogenic, as demonstrated by increased blood vessel density when patient-derived xenograft malignant epithelial cells were grown in vivo with senescent, versus pre-senescent fibroblasts^24^. Another study demonstrated that senescent glioma cells secrete proangiogenic SASP proteins including VEGF, resulting in increased tumorigenesis in vivo^9^. Of note, brain irradiation induces cerebrovascular injury, even in the absence of tumor^65,66^, which may foster a pro-angiogenic environment, thus potentially increasing tumor vascular arborization. The SASP also contains proteases, including matrix metalloproteinases, that degrade the microenvironment, facilitating tumor cell migration and invasion^25,67^. Importantly, the SASP can promote epithelial-to-mesenchymal transition^22^ (EMT), which is characteristic of highly aggressive tumors, including gliomas^68^. Indeed, one study found that partial removal of senescent (p16^Ink4a^) cells decreased the mesenchymal-like system and transcriptional subtype of the glioblastoma and its microenvironment, in vivo^54^. Whether this effect occurs directly in tumor cells or is mediated through tumor-associated monocytes remains under active investigation.

Another key deleterious effect of the SASP is induction of an immunosuppressive TME^69^ due to secretion of multiple SASP factors such as granulocyte-macrophage colony stimulating factor (GM-CSF) that can recruit and stimulate myeloid suppressor cells, as demonstrated in various non-glioma cancer lines^70^. The immune impacts of senescent cells is particularly important for cells that remain persistently senescent and do not escape senescence. These senescent cells can recruit other immune cells which can contribute to establishing and maintaining an immunosuppressive microenvironment^36^. Ablation of senescent cells and the immunosuppressive microenvironment could perhaps augment the efficacy of immunotherapies in glioma. Additionally, the SASP itself may induce further glioma development and proliferation. IL-6, a common SASP factor, is upregulated following chemoradiation and has been shown to be required for glioma development from glioma stem cells in a mouse model^71^, which may be of use for cancer cells that have acquired stemness after senescence induced by chemotherapy^8^.

While chemoradiation induces temporary senescence in glioma cells, we and others have shown that the brain irradiation-induced SASP, even in the absence of glioma cells, can contribute to glioma aggressiveness^13,72^. In one study, when mouse brains were pre-irradiated prior to tumor implantation (10 Gy), animal survival was significantly shorter than in non-pre-irradiated animals^13^. In contrast, irradiated p21-/- mice failed to exhibit radiation-induced senescence and did not promote tumor aggressiveness demonstrating that the senescent microenvironment was necessary to increase glioma aggressiveness. Although these and similar studies have been performed in multiple cell lines within immunocompetent models (GL261^13,73^, CT-2A^13^, NS2262^13^, and DBT^74^), we have observed a similar phenotype in six of ten glioblastoma patient-derived xenograft lines after 15 Gy of pre-irradiation^72^. Interestingly, pre-radiation extended survival in the two lines with the shortest time to moribund in vivo (GBM10 and 12), while survival in two other highly aggressive lines (GBM39 and 123) was not impacted by pre-radiation. These results may suggest that the SASP in the TME comprises a heterogeneous mix of factors, including some that are growth-promoting and others that are growth-inhibiting. Slower-growing tumor cells may be at higher risk of increased aggressiveness induced by the irradiated brain microenvironment. Of note, pre-irradiation-induced aggressiveness was seen both when glioblastoma cells were implanted at 48 h or 6 months after pre-irradiation^72^. This suggests that although senescence and SASP may evolve as a function of time since induction^40^, there is still a conserved pro-tumorigenic biological impact across time.

One limitation of such pre-irradiation studies to date has been that these studies pre-irradiate the non-tumor-bearing animals. To date, no study has directly compared the impact of an irradiated TME versus the irradiated non-tumor-bearing brain on recurrent tumor growth. The most clinically relevant scenario would be a previously irradiated tumor and its microenvironment, rather than a non-tumor bearing brain, as gliomas most often recur in the prior radiation field and not in radiation-naïve brain^2^. It remains to be seen how previously irradiated senescent tumor cells could impact the growth of non-senescent tumor cells. It also remains unknown how tumor cells that remain persistently senescent contribute to recurrence of other non-senescent glioma cells. However, human microdialysis has demonstrated that radiation induces a more robust proinflammatory response in tumor than brain^75^. As such, a previously radiated TME containing senescent tumor cells may contain even higher levels of SASP factors and induce more accelerated recurrent tumor growth.

### Astrocytes and microglia in the radiated brain

The impact of chemotherapy or radiation-induced senescence on endothelial cells and fibroblasts has been reviewed elsewhere^76,77^. However, unique components of the CNS tumor microenvironment include astrocytes and microglia. As previously noted, we and others found that radiation promotes senescence of astrocytes based on two independent markers of senescence (increased expression of p21 and loss of nuclear Lamin B1) in three different mouse strains (C57Bl/6 J, BALB/cJ, and FVB/NJ)^13^. One group also identified elevated markers of the senescence-associated proteins p16^INK4a^ and heterochromatin protein Hp1$\gamma$ in astrocytes from autopsy specimens from patients previously treated with radiation compared to non-treated autopsy controls^78^. Isolation of primary astrocytes, followed by irradiation or mock irradiation, confirmed increased SA-${\rm{\beta }}$-gal positivity and upregulation of multiple SASP-related genes by qRT-PCR. Irradiated astrocytes robustly secreted SASP factors including IL-1${\rm{\beta }}$, IL-6, and IL-8, and downregulated secretion of IGF-1, which is protective for neurocognitive function. Of note, radiation-induced astrocyte senescence could be rescued by overexpression of the p53 isoform, $\Delta$133p53, resulting in promotion of DNA repair, repression of IL-6 secretion, and increased IGF-1, suggesting that p53 is a central mediator of the paracrine influence of senescent astrocytes.

In addition to astrocytes, chemotherapy and radiation can induce senescence in microglia. Radiation is known to induce a pro-inflammatory phenotype in microglia through activation of NF-${\rm{\kappa }}$B and mitogen-activated protein kinase (MAPK) pathways, resulting in increased cytokine secretion and neuronal apoptosis^79^. Radiation also induces senescence in tumor-associated macrophages (TAMs)^80^, while aiding in their recruitment in addition to that of activated peripheral monocytes and other immune cells^81^, both in non-glioma cancer models. Of note, the relative burden of therapy-induced senescence as a percentage of each cell population has yet to be determined, particularly in patient-derived specimens. It also remains to be elucidated if and how each cell type’s therapy-induced senescence may contribute to glioma aggressiveness, including SASP components produced by each human cell type in vivo, and the impact thereof on glioma invasion and proliferation.

### The senescent escape

In addition to the deleterious effects of the SASP, therapy-induced senescence may allow glioma cells to escape apoptosis, thereby contributing directly to risk of recurrence. An in vitro study demonstrated that knockdown of *survivin* induced senescent U251 cells to become apoptotic, increasing the sensitivity of these cells to TMZ^82^, suggesting that tumor senescence can be used as an escape from therapy-induced apoptosis. While senescence has typically been considered a state of irreversible cell cycle arrest, multiple studies in non-glioma cancer models have demonstrated that senescent cancer cells can re-enter the cell cycle^8,10–12,82^. Whether distinct molecular profiles distinguish senescent versus dormant cancer cells is an important unanswered question. Unfortunately, senescence appears to induce permanent epigenetic reprogramming of cancer cells leading to increased stemness^8^. Upon re-entering the cell cycle, these cells have increased potential for tumor initiation in vivo and greater proliferative capacities in a WNT-dependent manner^8^. Additionally, data in other cancer types suggest that stemness-associated transcripts are highly enriched in recurrent malignancies^83,84^. This may occur due to de-repression of the telomerase reverse transcriptase gene^85^, particularly in the early stages of tumor progression. It is important to note that escape of senescence likely does not need to occur in all senescent cells for these to contribute to recurrence. Rather, as demonstrated in prior non-glioma studies^8,10–12,82^, we speculate that only a small subset of senescent cells escape latency; once they escape, their uncontrolled proliferation could then contribute to glioma recurrence. Potential mechanisms of senescence escape have been extensively reviewed elsewhere^12^. Unfortunately, few such studies to date have focused on glioma, likely due to the absence of available longitudinal tissue sets that include primary tumor, latent disease, and recurrent glioma from individual patients.

While glioma stem cells are thought to contribute to tumor recurrence^86^, perhaps by activating DNA damage checkpoint responses during radiation^87^, no study to our knowledge has directly determined if those stem cells maintained their stem-like behavior throughout therapy. We posit that the tumor-initiating stem cells observed after therapy likely passed through a senescent-like state of proliferative arrest prior to re-emergence as tumor stem cells. Alternatively, prior in vitro glioblastoma studies suggest that senescence is only induced in a subset of tumor cells, ranging from 60–80% at the time of maximal senescence burden to about 10% at later timepoints^88^. It is thus also possible that there may be a subset of chemoradiation-resistant glioma stem cells, or non-stem cells, which were never senescent and contributed to recurrence (previously reviewed)^89^. In summary, the glioma microenvironment after treatment is likely a complex microenvironment composed of (1) senescent tumor cells, a minor subset of which have the potential to escape senescence, along with (2) glioma non-stem and stem cells that resist chemoradiation—all of which could contribute to recurrence. The sustained presence of treatment-induced senescent cells in the microenvironment (as reviewed in “The senescent tumor microenvironment (TME) impacts glioma aggressiveness”) could lead to the ongoing release of SASP, thereby potentially elevating the aggressiveness of any of these cell types upon recurrence. Future studies will be needed to directly test the relative contributions of senescence-escape versus inherent chemoradiation-resistance to glioma recurrence.

### Senescence and therapy-induced adverse effects

Senescence has been heavily implicated in cognitive decline, which has previously been reviewed^90,91^. Indeed, we and others have demonstrated that clearance of senescent cells, either pharmacologically or genetically, ameliorates cognitive functioning in preclinical models^14,15^. Radiation and chemotherapy are known to adversely impact cognition in patients^92^. Prior studies have demonstrated that even low-dose radiation exposure transcriptionally alters pathways related to cognitive functioning and advance aging^93^. Additionally, whole-brain irradiation increases the number of senescent astrocytes and impairs astrocytic calcium signaling^94^. While whole brain radiation therapy (WBRT) is no longer as widely utilized for patients with gliomas, patients continue to experience neurocognitive decline when undergoing chemotherapy and radiation^95^, perhaps in part due to the far-ranging inflammatory impacts of the SASP^47^.

Senolytics could ameliorate these therapy-induced adverse effects. Indeed, treatment with navitoclax, a Bcl-2, Bcl-xL, and Bcl-W inhibitor, improved cognitive performance after clearing senescent endothelial cells and improving blood-brain barrier integrity in a mouse model of chemotherapy-induced cognitive deficits^96^. Senescent cell clearance may also improve neurovascular coupling, an important contributor to cognitive function^97^. Other studies in non-glioma diseases have found that navitoclax and dasatinib + quercetin, a senolytic combination, improved short-term memory, both when senolytics were given prior to and after induction of radiation-induced cognitive deficits^98,99^. As such, targeting of the senescent glioma and its microenvironment may enable further alleviation of the detrimental cognitive impacts of standard-of-care treatment.

## Senolytics: an opportunity to target therapy-induced vulnerabilities?

Senolytics may be of particular relevance in glioma to (1) ablate latent tumor cells within the treated field that could otherwise contribute to tumor recurrence, (2) decrease the abundance of pro-tumorigenic SASP factors in the TME, decreasing the amount of “fertilizer” available for glioma recurrence, and (3) alleviate senescence-induced cognitive and bone marrow decline from prior treatment. Given that no other effective drugs are available to patients after completion of adjuvant TMZ, senolytics are promising candidate therapeutics that could be administered in the latent stage to delay or prevent recurrence (Table 1).

### Candidate senolytic agents in glioma

#### Inhibition of anti-apoptotic Bcl-2 family proteins

Bcl-2 family protein inhibitors are a major class of candidate senolytics that target the anti-apoptotic Bcl family, including Bcl-2, Bcl-xL, and Bcl-W^100^. To date, most relevant preclinical data related to glioma and CNS senescence have been generated using ABT-263 (navitoclax), which targets these three Bcls^101^ and is currently being studied in hematologic malignancies including chronic lymphocytic leukemia (CLL) and myelofibrosis. Multiple in vitro studies have demonstrated that ABT-263 induces apoptosis in senescent glioblastoma cells at a lower IC50 than non-senescent glioma cells. To our knowledge, this observation has been consistent across every grade 4 astrocytoma cell line reported to date (both IDH-wild type and mutant), including LN229^102^, A172^102^, IDH1-R132H mutated U87MG^103^, and 9 molecularly diverse human lines spanning P53 and IDH-WT primary and recurrent lines from male and female patients^104^. However, sensitivity to Bcl-xL inhibition varied widely by cell line—both before and after radiation^104^. In vivo treatment with ABT-263 in the pre-irradiated glioma model ablated senescent astrocytes, decreasing the aggressiveness of glioblastoma cells implanted into the previously radiated brain^13^. Similarly, ABT-263 prolonged survival in an in vivo U87MG IDH1-R132H model, which was replicated in an IDH-mutant GBM164^103^. Similar results were also obtained with the similar multi-Bcl-targeting compound ABT-737^102^. Use of anti-apoptotic Bcl-2 family protein inhibitors could also promote anti-inflammatory effects that could otherwise contribute to glioma recurrence^105^.

Unfortunately, use of navitoclax in both preclinical models and patients is limited by drug toxicities, including thrombocytopenia^106^ that decrease its long-term tolerability and utility for CNS malignancies wherein hemorrhage could be catastrophic. Periodic short, high-dose senolytic regimens would likely be more tolerable by patients and has been effectively utilized in patients^53^. However, extended treatment with relatively high doses has generally been required to ablate senescent cells from the CNS^13,103^, increasing the risk of unacceptable toxicities. Additionally, while the relative CNS penetration of ABT-263 remains unknown, the prolonged exposure that is sometimes necessary for efficacy may prove unfeasible given toxicities, nor has the potential for intrathecal or direct CNS delivery been evaluated in patients. Bcl-xL selective proteolysis-targeting chimeras (PROTACs) may have less off-target toxicities^107^, although their relative CNS penetration and effect in gliomas remains unknown.

Other Bcl-2 family protein inhibitors include venetoclax (Bcl-2 inhibitor), and the model compounds A1331852 (Bcl-xL inhibitor), and A1155463 (Bcl-xL inhibitor). We have previously shown that the selective Bcl-xL inhibitors A1331852 and A1155463 induced preferential apoptosis of senescent human glioma cells in a similar manner to navitoclax^104^. This result could not be reproduced with the FDA-approved selective Bcl-2 inhibitor, venetoclax, suggesting that senescent glioma cells are relatively dependent upon Bcl-xL for survival. In contrast, another group found that utilizing venetoclax 120 h after an initial round of TMZ significantly increased cell death from 26% to over 60% in LN229 cells^108^. Interestingly, in our studies^104^, the senolytic impact of Bcl-xL inhibition was maximally seen by 96 h post-radiation, as compared to 24 h after radiation, suggesting that senolytic therapies could be effective relatively soon after DNA-damaging therapy. This also suggests the importance of considering how senescence may evolve over time when evaluating senolytics. Further work is needed to evaluate the impact of senolytics at much later timepoints after induction of senescence. In summary, while Bcl-2 family targeting agents are available clinically as candidate senolytic agents, concerns for toxicity, questions about CNS penetration, and variable sensitivity in tumors represent potential challenges to clinical translation.

#### Flavonoids

Flavonoids, such as quercetin, are phytochemical compounds found in plants that have become some of the best-known candidate senolytics. Quercetin, when combined with dasatinib, has been shown to reduce senescent cells across multiple cell types and disease states, including degenerative disc^109^, diabetic kidney^53^, and neurodegenerative diseases^15^. Dasatinib and quercetin target different SCAPs, including tyrosine kinases^110^ (dasatinib) and the PI3K/AKT/mTOR^111^, p53/MDM2/p21^112^, and HIF1${\rm{\alpha }}$-related pathways^113^ (quercetin). Dasatinib and quercetin (D + Q) are currently in clinical trials as a senolytic cocktail, with initial results in diabetic kidney disease demonstrating clearance of senescent cells in adipose tissue and decrease in circulating SASP factors^53^. While studies assessing D + Q have been promising in other disease states, our in vitro results from GBM39 cells did not demonstrate a substantial impact of dasatinib, quercetin, or D + Q as a senolytic combination following irradiation^104^. However, other groups have found that fisetin, another flavonoid, exhibited significant senolytic activity after TMZ-induced senescence in LN229 and A172 cells^102^. In contrast, we did not find a significant impact of fisetin on senescent cell death in vitro after irradiation of GBM6, 39, or 76 cells^104^. Given the promising results to date in other diseases and the highly favorable safety profile of flavonoids, along with a relatively low cost, further studies are warranted.

The senolytic impact of these flavonoids could be dependent on components of the microenvironment not represented in a culture dish, such as the resident and peripheral immune cell population, perhaps explaining why in vitro and in vivo results with senolytics have sometimes conflicted. Indeed, these flavonoids induce clearance of senescent microglial cells^15^ and reduce trafficking of circulating monocytes and macrophages to senescent tissue^53^, which could decrease the presence of deleterious SASP components in the TME. As such, there is reason to speculate that flavonoids could yield therapeutic impact within the tumor ecosystem. Moreover, given the safety of these compounds, early phase 0 studies in patients with gliomas may enable individualized “go or no go” answers regarding the utility of senolytics as proposed in “How to translate senolytics into the clinic?”.

#### Targeting the inhibitors of apoptosis family

Another class of candidate senolytics target inhibitors of apoptosis protein (IAP) family. One study has found that c-IAP2 was upregulated within 120 h of TMZ exposure in LN-229 cells, concurrently with induction of senescence^108^. Treatment with BV6, a small molecule that targets c-IAP1 and c-IAP2, has been shown to selectively ablate the senescent LN229 and A172 cells in vitro following TMZ^102^. Interestingly, the combination of BV6 and venetoclax (Bcl-2 inhibitor) with TMZ increased cell death from 65% with BV6 + TMZ or 60% with venetoclax+TMZ to over 80% with BV6+venetoclax+TMZ^108^. Additionally, for the cells that escaped senescence and regained their proliferative properties after TMZ, two of the three TMZ-resistant clones retained sensitivity to BV6. This suggests a continued role for c-IAP2 in tumor therapeutic resistance, and contrasts with our preliminary observations that re-entering cell cycle attenuated sensitivity to Bcl-xL inhibition^104^. In vivo studies are needed to evaluate the senolytic utility of c-IAP inhibitors for glioma.

#### Other candidate senolytics and SASP-targeting agents

Artesunate is an antimalarial agent that is under preclinical evaluation for repurposing as a senolytic agent. Though not well defined, artesunate likely functions through multiple mechanisms of action^114^. In vitro studies demonstrated selective pro-apoptotic senolytic activity in TMZ-treated LN229, A172, and U87MG cells, as well as glioblastoma stem-like cells (G112SP)^102,115^. In vivo data in senescent glioma models are needed. Of note, intravenous artesunate is generally well-tolerated in patients with malaria, with rare side effects including allergic reactions and hemolytic anemia^114^.

Heat shock protein 90 (Hsp90) inhibitors, including onalespib and 17-DMAG, demonstrated senolytic activity in senescent embryonic fibroblasts^116^. 17-DMAG also decreased senescent cell burden in a progeroid mouse model^116^, although its impact in glioma remains unknown. Combined treatment with onalespib and TMZ extended survival in U251 and GS811 mouse intracranial xenografts as compared to TMZ alone^117^. In line with these findings, we observed the cytotoxic impact of onalespib in GBM6, 39, and 76, in vitro^104^. However, the IC50 was not impacted by prior radiation, suggesting that this drug may not be a true senolytic agent in glioma cells. Nevertheless, onalespib crosses the blood-brain barrier^117^ and has undergone phase 1 trials with acceptable safety and toxicity findings^118^.

#### Targeting individual SASP components

Although not directly a senolytic, strategies to decrease SASP production (senomorphic) could help mitigate the deleterious impacts of senescent cells. As previously described in “The senescent tumor microenvironment (TME) impacts glioma aggressiveness” and “The senescent escape”, IL-6 is one of the most important and pro-tumorigenic factors in SASP^71,119,120^. Given its role in facilitating a glycolytic phenotype and glioma survival and proliferation, IL-6 is an interesting target for glioma therapy. Since IL-6 promotes gliomagenesis and it is a known SASP factor, blocking IL-6 could help attenuate tumor growth in the previously radiated microenvironment. To date, addition of tocilizumab, an IL-6 therapeutic monoclonal antibody, decreased growth of TMZ-treated subcutaneous U251 glioblastoma models^121^. In another study, targeting IL6R-alpha or IL-6 with short hairpin RNAs reduced the growth and neurosphere-forming capacity of glioma stem cells (GSC)^71^. In vivo, IL-6 or IL6-R knockdown in tumor cells significantly increased survival in intracranial T3359 GSC models.

An alternate approach could be to target downstream signaling pathways of SASP components. Fletcher-Sananikone et al. found that both radiated astrocytes in vitro and radiated brain upregulated HGF expression^13^. Blocking Met downstream of HGF with crizotinib mitigated the pro-migratory impact of senescent astrocytes in vitro and modestly improved survival in a pre-radiated GL261 glioblastoma model in vivo^13^.

Finally, while not yet utilized in glioma models, senolytic chimeric antigen receptor (CAR)-T cells have been developed that are specific to a surface marker of senescent cells, urokinase type plasminogen activator receptor (uPAR)^122^. These CAR-T cells have demonstrated a promising ability to ablate senescent cells in vitro and in vivo, although the survival impact of this therapy in preclinical glioma models has yet to be demonstrated. Given the role of senescence in promoting a tumor-suppressive microenvironment, combinatorial use of senolytics with a checkpoint inhibitor could be useful to augment the efficacy of the immunotherapy by clearing the tumor-immunosuppressive microenvironment via ablation of senescent cells^69^.

In conclusion, multiple candidate senolytic agents exist for potential use in glioma, although a major limitation of many of these studies is lack of in vivo validation, raising concerns that different results could be obtained in the more complex senescent tumor microenvironment—if the drugs are able to reach the tumor at adequate levels without unacceptable systemic toxicity. It also remains unknown how the mouse senescent microenvironment compares to that of the human one. Prior reviews would suggest that there will be significant differences between the human and murine TME^123^. As such, attempts to extrapolate the therapeutic impacts in preclinical models to humans should be interpreted with caution.

### 3.2 Anticipated challenges and unanswered questions regarding glioma senolytic therapy

Although senolytics may present an interesting and untapped therapeutic avenue, multiple questions and challenges remain: (1) the optimal agent(s), dose(s), timing, and cycling of senolytic therapies are unknown, and may be challenging to accurately infer from preclinical data, (2) enrolling patients for clinical trials in the presence of latent disease would be a paradigm shift for glioma, and (3) relevant outcome measures should be identified to evaluate the efficacy of therapy, including blood or CSF biomarkers.

#### Limited understanding of latent human glioma

The latent human glioma and its microenvironment remains largely unexplored. To help address the current dearth of knowledge about latent human gliomas, we have opened a prospective single-arm trial for surgical resection of latent brain tumors prior to recurrence (NCT04810871). Tissue obtained from this trial will help address gaps in knowledge including: (1) the relative frequency, identity, and heterogeneity of senescent cells among different cell populations in the TME, (2) the relative contribution of each senescent cell population to SASP production, (3) the impact of senescent cells on the aggressiveness of human glioma cells that have escaped senescence to re-enter the cell cycle, and (4) the relative vulnerability of senescent glioma and stromal cells in situ to candidate senolytic agents. To help address these and other questions, tissue harvested during this trial is cryopreserved to enable functional studies and ex vivo assays evaluating sensitivity to senolytic drugs or cocktails.

#### Senolytic dosing approaches to senescent latent glioma: the “one-two” punch hypothesis

The “one-two” punch hypothesis suggests that senolytics should be used after senescence has been induced by a primary therapy^5,124^. This concept was first demonstrated when multiple melanoma and lung cancer cell lines acquired sensitivity to ABT-263, a senolytic targeting Bcl-2, Bcl-xL, and Bcl-W, and has since been utilized in various other cancer models^125^. This method should limit combination-associated toxicities and may help attenuate resistance to standard-of-care therapies^17,126^. For this approach to be successful, most, but perhaps not all, senolytics would be optimally administered prior to tumor cells escaping from their senescent-like state of mitotic arrest.

A population of senescent cells may be present throughout disease, beginning even prior to cytotoxic therapy^54^. Nevertheless, it would stand to reason that the highest percentage of tumor cells will be senescent during the latent disease period following completion of chemoradiation. As recurrence of some gliomas, including those that are MGMT methylated^57^ and/or IDH-mutant^127^, can be delayed for many months to years, senolytics could be used during this period of latency to serve a second “punch” against therapy-induced senescent cells. However, it remains untested whether it is best to wait until completion of therapy to begin senolytic therapy or if senolytics should be started during standard-of-care cytotoxic treatment when senescence is being induced. The optimal duration and frequency of therapy may need to be determined empirically in carefully designed early phase clinical trials that provide mechanistic feedback.

Of note, our in vitro data suggest that a small percentage of senescent cells may remain after treatment with senolytics^104^, suggesting that multiple rounds of senolytics perhaps targeting different SCAPS sequentially or in combination may be required to clear these cells. Longitudinal sensitivity to senolytics, and potential resistance, would need to be considered. After chemoradiation, it remains unknown if senolytic sensitivity could be augmented by rechallenge with further senescence-inducing therapy. Options could include TMZ or lomustine, which are both FDA-approved therapies utilized in patients with glioma. Other therapies could include Tumor-Treating Fields (TTFs)^128^, which is an FDA-approved alternating electrical field therapy that is utilized by some, but not all patients with high-grade gliomas, as it requires wearing a device for ideally 18 h a day or more. Preliminary data in mesenchymal stromal cells suggest that TTFs may induce senescence^129^, although the senescence-inducing impacts of this therapy remain to be evaluated within in vivo gliomas. Currently, most senolytic studies have a cyclical dosing schedule, wherein they are administered at high doses for a few days in 28-day cycles^50^. This may fit conveniently into the standard Stupp protocol that includes TMZ on the first 5 days of each 28-day cycle following radiation^1^. Alternating cycles of TMZ and senolytics every 2 weeks, either during the adjuvant phase of treatment and/or in latency, could help promote senolytic sensitivity while minimizing theoretical risk of toxicity from concurrent medications. Systematically identifying agents to augment senolytic efficacy may prove a useful next step for the field, allowing senolytics to be effective at lower and less toxic doses that may be more feasible to achieve in the context of the blood-brain barrier.

#### Paradigm shift: enrolling patients in clinical trials without active disease burden?

There are currently no therapeutic trials for patients with latent glioma. The standard clinical trial pipeline tests agents in the context of active disease—be it primary or recurrent disease. Enrolling patients into therapeutic trials during the latent stage of disease is rarely ever performed in neuro-oncology. Nevertheless, given the deleterious impacts of senescence and its SASP, latent glioma is an untapped opportunity to evaluate agents that could delay the time to and probability of recurrence and improve quality of life, perhaps including progression to higher tumor grades. There are no available therapeutic drugs during this period. In our experience, many would be motivated to actively pursue clinical trial opportunities, if available, rather than wait for inevitable recurrence. As virtually all glioma patients will have latent disease at some point during their clinical course, there is no paucity of potential patients for consideration. Furthermore, recent prolongation of progression-free survival with vorasidenib, an IDH inhibitor, in patients with residual or recurrent IDH-mutant gliomas, may further increase the number of patients with latent disease for senolytic clinical trials^130^.

#### Monitoring the efficacy of senolytic and senomorphic therapies

No imaging strategy or laboratory study has been developed to specifically quantify senescent CNS cell burden. Some patients may have little, if any, evidence of residual disease after maximal safe resection and chemoradiation. As such, novel methods will be needed to quantify evidence of latent tumor cells and/or SASP factors to determine whether or not senolytic therapy is successfully decreasing disease burden. Ongoing senolytic studies evaluate plasma, serum, and urine levels of SASP factors^47^, looking for declines after senolytic therapy^53^. However, given the presence of the blood-brain barrier, cerebrospinal fluid (CSF) could serve as a more reliable source of biomarkers originating from latent gliomas or prior resection cavities^131^. Assaying CSF for SASP, or other candidates like mitochondria-derived nucleotides that are elevated in senescence^132^, would thus be of high utility for longitudinally monitoring the impacts of senolytics on the relative senescent cell burden. Longitudinal monitoring of SASP via CSF could also be utilized to understand how senescence and SASP evolve as a function of time since chemoradiation in gliomas, without the use of senolytics. Of note, quantitative assays for SASP factors and biomarkers of latent glioma cells will need to be independently developed to concurrently assess the burden of total senescent cells, and residual tumor cells, respectively.

## How to translate senolytics into the clinic?

To date, no senolytic drug or cocktail has been evaluated in patients with glioma during the latent stage of their disease, when tumor cells would be presumably most vulnerable. The latent stage of each patient’s disease is an untapped opportunity for the evaluation of candidate senolytic agents. Senolytics could be translated to the clinic in traditional phase 1+ trials to establish their safety and tolerability, followed by their efficacy in later phase trials. A limitation is that such trials may take a significant amount of time to complete, particularly for patients with lower grade tumors that may have years of latency prior to the tumor’s inevitable recurrence. Most importantly, based on the potential for heterogeneity in senescence across patients and within tumors, phase 1+ trials would not provide rapid, patient-individualized feedback to determine whether a candidate senolytic agent is effective for that individual’s tumor and its microenvironment.

**Fig. 2:**
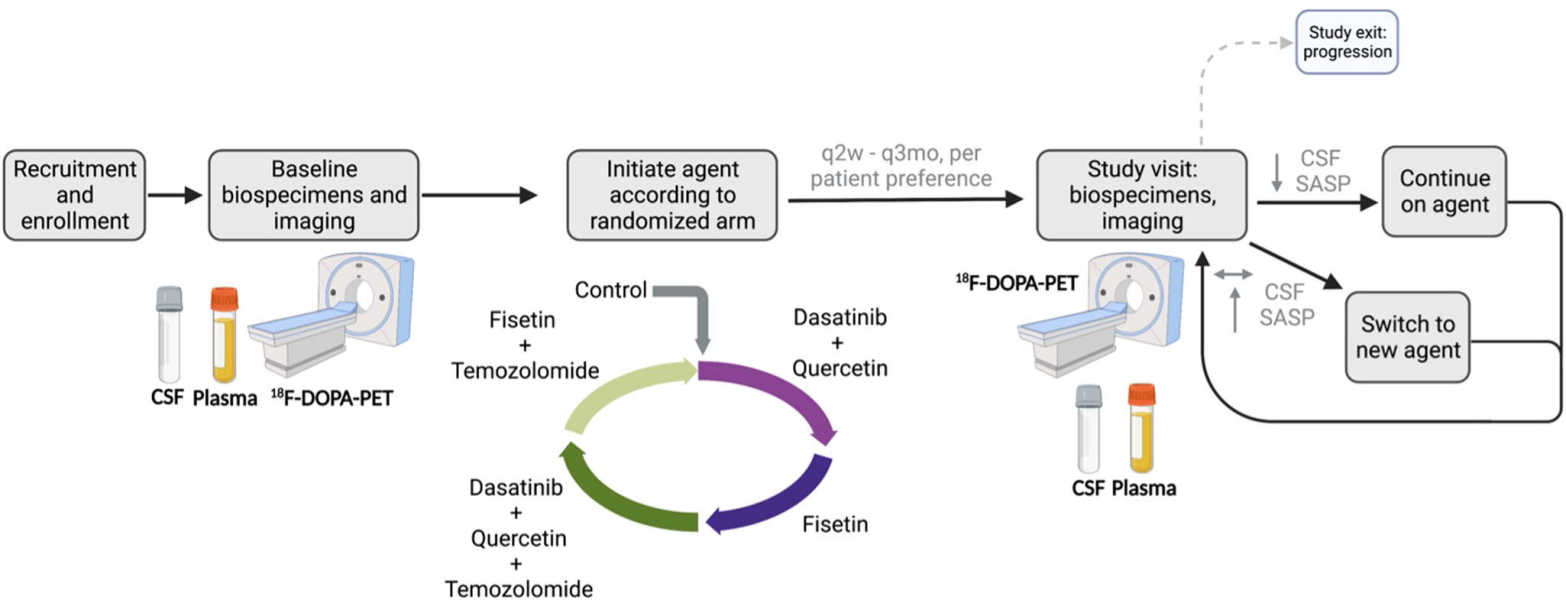
Candidate clinical trial design for latent glioma. In patients with latent glioma and CSF access devices, baseline biospecimens and imaging would be acquired prior to initiation of a sequence of senolytics. Biospecimens and imaging would be acquired every 2 weeks to 3 months to assess for pharmacodynamic response based on the CSF SASP. Lack of response would lead to discontinuation of that agent and initiation of the next one in the sequence.

To that end, phase 0 studies for senolytics in pre-recurrent gliomas could empower identification of pharmacodynamic biomarkers in response to candidate senolytic agents based on acquisition of longitudinal data, including serial CSF samples from Ommaya reservoirs implanted at the time of surgery. We ultimately aim to develop a panel of CSF senescence-related biomarkers, in addition to plasma and imaging modalities, to determine whether a patient continues or transitions to a new therapy. Toward that goal, initial discovery efforts will be required to (1) identify signatures of senescence and SASP within CSF and (2) understand the pharmacodynamic impact of candidate senolytics on CSF, plasma, and imaging. To initially deploy this phase 0 pharmacodynamic paradigm, flavonoids, such as quercetin or fisetin, could be utilized as they have a highly favorable safety profile and are currently in use in other clinical trials^53^. TMZ could be utilized to help upregulate SCAPs and maximize chances of glioma cells being in a senescent-like state at the time of senolytic therapy. Although the use of pharmacodynamic response based on SASP is currently discovery-based and speculative, an example protocol is illustrated in Fig. 2.

A limitation of the phase 0 approach via CSF is that it would remain unknown how changes in CSF SASP correlate with senescent cell burden—some of which may be produced by both senescent cells and the inflammatory stroma of treatment-naïve glioma. Nevertheless, sequential evaluation of senolytics in this manner could provide an opportunity to efficiently evaluate the individualized impact of multiple senolytics, sequentially, on multiple candidate biomarkers of senescence, based on the relatively rapid mechanism of action of senolytics^7^. Ultimately, further in vivo studies with senolytics and senomorphics are needed to evaluate the impact of these agents in latent glioma, which is currently an untapped window of therapeutic opportunity in the translational pipeline prior to disease recurrence.

## Conclusions

Chemoradiation induces senescence in gliomas and their TME, contributing to disease latency and inevitable recurrence. This senescent state provides an attractive therapeutic target to (1) prolong the time to recurrence by ablating latent senescent cells, (2) deplete SASP components released by senescent cells and hopefully decrease the aggressiveness of future recurrence, and (3) mitigate the long-term impacts of senescence on neurocognitive functioning and bone marrow function in patients with glioma. Multiple candidate senolytic agents, including anti-apoptotic Bcl-2 family inhibitors and flavonoids, currently exist that may be leveraged to decrease the senescent cell burden left in the wake of chemotherapy and radiation. There remain many outstanding questions regarding the potential efficacy of these agents, their optimal administration, and biomarkers of efficacy. However, patients with latent gliomas have few therapeutic options after chemoradiation prior to recurrence, providing a unique opportunity to evaluate senolytics in situ through phase 0, algorithm-based studies, which could lead to the development of novel therapeutic strategies to prevent glioma recurrence.
